# Idiopathic retroperitoneal fibrosis with endometrial cancer: a case report and literature review

**DOI:** 10.1186/s12905-022-01968-8

**Published:** 2022-10-01

**Authors:** Manfei Si, Kun Zhang, Jiaxin Li, Huiying He, Ying Yao, Jinsong Han, Jie Qiao

**Affiliations:** 1 Center for Reproductive Medicine, Department of Obstetrics and Gynecology, Peking University Third Hospital, Beijing, 100191 China; 2 National Clinical Research Center for Obstetrics and Gynecology, (Peking University Third Hospital), Beijing, 100191 China; 3 Key Laboratory of Assisted Reproduction (Peking University), Ministry of Education, Beijing, 100191 China; 4 Beijing Key Laboratory of Reproductive Endocrinology and Assisted Reproductive Technology, Beijing, 100191 China; 5 Department of Obstetrics and Gynecology, Peking University Third Hospital, Beijing, 100191 China; 6 Department of Pathology, Peking University Third Hospital, Beijing, 100191 China

**Keywords:** Retroperitoneal fibrosis, Endometrial cancer, Enlarged lymph nodes, Tamoxifen, Case report

## Abstract

**Background:**

Retroperitoneal fibrosis is a rare disease characterized by chronic nonspecific inflammation, which leads to clinical compression manifestations of retroperitoneal organs especially ureter. Approximately 70 percent of retroperitoneal fibrosis cases are idiopathic which has no clear etiology. This study reported a rare case of a 48-year-old woman presented with idiopathic retroperitoneal fibrosis and endometrial cancer.

**Case presentation:**

A 48-year-old woman presented with irregular vaginal bleeding without abdominal pain, bloating or discomfort. The patient was diagnosed iRPF after splenectomy 13 years ago. Then she took prednisone for 2 years and took tamoxifen for about 11 years. She stopped taking the medication from October 2019 to May 2020 and then started taking tamoxifen again until November 2020. Two weeks after she stopped taking tamoxifen, she presented with irregular vaginal bleeding. Gynecological ultrasound revealed a thick endometrium with uneven echo enhancement and blood flow signals. Then diagnostic curettage was performed with pathological examination showed endometroid carcinoma. Later, the patient was admitted to Peking University Third Hospital for surgery. Preoperative imaging examinations, including CT, MRI, and PET/CT, all showed pelvic enlarged lymph nodes and they were highly suspected to have lymph node metastasis. The patient underwent laparoscopic surgical staging and enlarged lymph nodes in the pelvic and aortic regions were removed. Finally, the pathology confirmed that endometrioid adenocarcinoma and fibrosis, but there was no tumor infiltration in these enlarged lymph nodes. The patient is now in good condition.

**Conclusion:**

This case report stressed the difficulty to distinguish between lymph node metastasis and inflammatory hyperplasia by common imaging methods. Due to increased surgical difficulty among retroperitoneal patients, lymphadenectomy should be carefully evaluated to avoid additional surgical complications and over-treatment.

## Background

Retroperitoneal fibrosis (RPF) is a fibroinflammatory condition clinically characterized by fibrotic retroperitoneal mass around abdominal aorta and the iliac arteries. Most RPF cases are idiopathic (iRPF) and the others are secondary to malignancies, infections, drugs, etc. Goals of RPF treatment are to relieve the compression symptoms and to prevent the progression of inflammation and fibrosis. Glucocorticoid is currently considered to be the first-line medication for RPF [1–3]. Besides glucocorticoids, tamoxifen is also reported to be a suitable alternative which is safe and effective [4, 5]. Previous studies did not report that RPF patients suffered from an increased risk of developing endometrial cancer when taking tamoxifen. In this case report, a rare case of a woman presented with iRPF and endometrial cancer was described, and this emphasized a potential risk of long term use of tamoxifen therapy on the development of endometrial cancer for female iRPF patients. This case also stressed on the difficulties in distinguishing between inflammatory fibrosis and malignant infiltration of lymph nodes in diagnosis.

## Case presentation

### Medical history

A 48-year-old woman presented to the gynecology clinic with irregular vaginal bleeding that started one month ago (in November 2020). Her regular period had stopped in 2016 after adding cyclophosphamide to treat iRPF, and this completely stopped her menstrual cycle until irregular vaginal bleeding was observed after the removal of tamoxifen from her treatment in 2020. The patient reported no abdominal pain, bloating, discomfort, or other urologic manifestations such as frequency urination, hematuria, and dysuria. She also had no history of obesity (body mass index with 20.7 kg/m^2^), diabetes, hypertension, or family history of cancer, but a long-term use of tamoxifen for almost 11 years. The patient had a 13-year history of iRPF which was diagnosed in Peking Union Medical College Hospital after splenectomy, which was performed following symptoms of fever, increased abdominal pain accompanied by anemia, splenomegaly, and multiple enlarged lymph nodes in abdominal cavity and retroperitoneum. Her right kidney atrophied and presented with hydronephrosis in unison. Laboratory tests showed hemoglobin (Hb) 87 g/L, erythrocyte sedimentation rate (ESR) 57 mm/h, and antinuclear antibody (ANA) positive 1:160. After surgery, she began taking prednisone and tamoxifen on doctor’s advice. During the period of using prednisone, the patient received regular monitoring follow-ups including inflammatory indicators including ESR and C-reactive protein (CRP) and imaging (ultrasound). These indicators were stable thus the dosage of prednisone was gradually reduced and stopped after 2 years in order to avoid side effects (in 2010). Since 2011, the patient was prescribed tamoxifen alone for maintenance treatment of iRPF. In 2016, her imaging showed multiple retroperitoneal lymph node enlargement and the inflammatory indicators (ESR and CRP) were elevated. In addition to tamoxifen, cyclophosphamide was added to prevent the progression of iRPF. She stopped taking her medication all together from October 2019 to May 2020 as a personal choice. Then she started taking tamoxifen again until November 2020 when the endometrium was found thick. Two weeks later she stopped taking tamoxifen, she presented with irregular vaginal bleeding for 20 days without abdominal pain. Physical examination of the uterus and bilateral appendages was unremarkable. Transvaginal gynecological ultrasound revealed a thick endometrium (11 mm) with uneven echo enhancement and some blood flow signals, and there was no clear boundary between the endometrium and myometrium. Multiple fibroids with clear borders were detected in the uterus, and the blood flow signal was not rich. No ascites, pelvic fluid or other imaging abnormalities were found. Additionally, the levels of tumor markers such as CA125, CA199, CEA, and NSE were all within the normal range. Therefore, diagnostic curettage was performed, and pathological examination of endometrium showed well-moderately differentiated endometroid carcinoma and it could be classified in POLE hypermutation subgroup based on the TCGA molecular classification. Also, the immunohistochemistry (IHC) results showed P53 (wild type), MLH1-positive, MSH2-positive, MSH6-positive and PMS2-positive. Later, the patient was admitted to Peking University Third Hospital for surgery.

### Clinical procedures

**Fig. 1:**
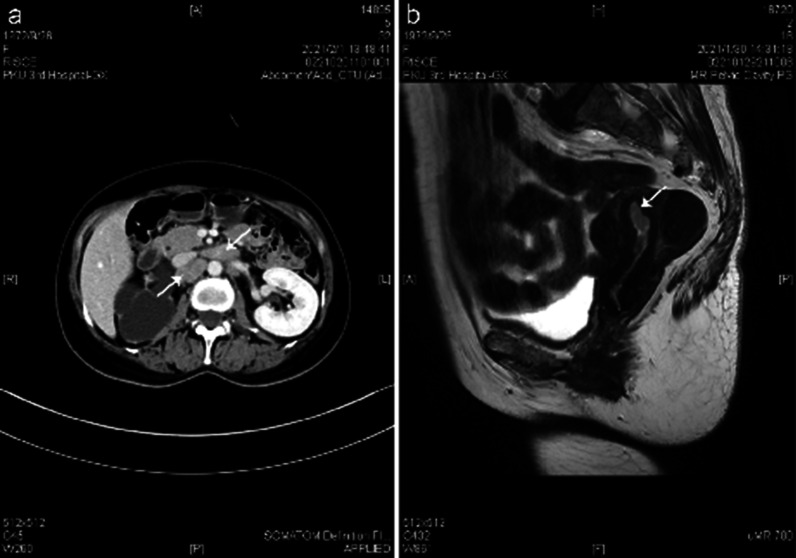
Imaging findings of CTU and MRI. **a** The arrows pointed to the enlarged lymph nodes. **b** The arrow pointed to thickened endometrium

**Fig. 2:**
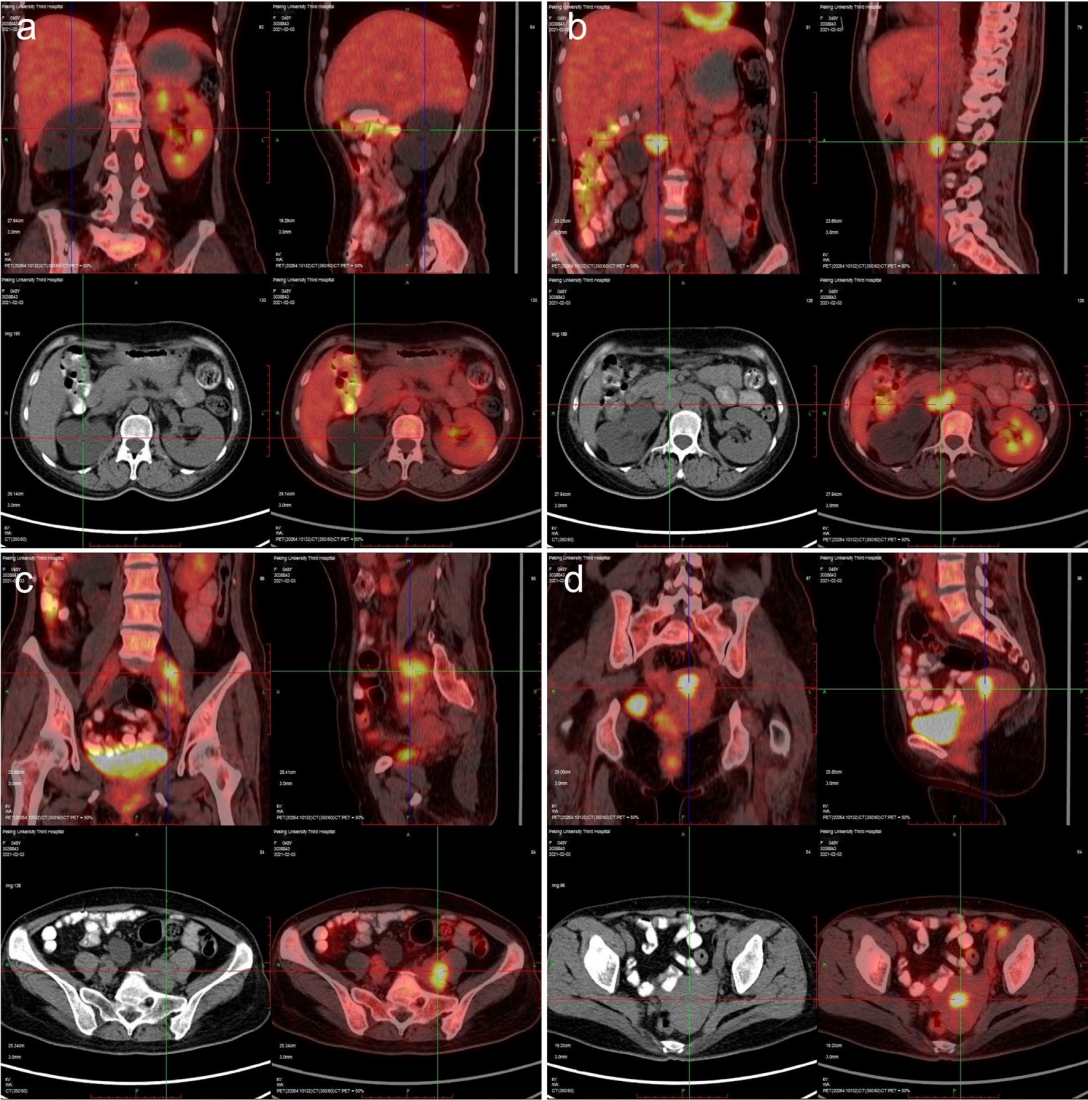
PET/CT findings of various organ involvements in this patient were presented. **a** Renal involvements. The right renal parenchyma was thinned, associated with dilation and hydronephrosis of the right renal pelvis and ureter. **b** Multiple enlarged lymph nodes with increased FDG uptake in the retroperitoneum, the largest one was about 3.3 × 2.5 cm. **c** Multiple enlarged lymph nodes with increased FDG uptake in the bilateral iliac vascular areas, the largest one was located on the left side, about 3.4 × 2.0 cm. **d** Patchy radioactive focus in the uterine cavity

The results of preoperative laboratory tests are presented below (Table 1). Both CT and MRI revealed thickened endometrium and multiple enlarged lymph nodes in the pelvis, which suggested great possibilities of endometrial cancer with lymph node metastasis (Fig. 1). Abdominal ultrasound showed severe right hydronephrosis and right ureter dilatation. CT urography (CTU) enhancement demonstrated right urinary tract dilatation, right hydronephrosis and hydroureter, right kidney atrophy and stenosis of the right lower ureteral lumen. Her right kidney function was severely impaired, but the left kidney compensated enough so her renal function was normal seen under the renal dynamic imaging. PET/CT revealed increased fluorodeoxyglucose (FDG) uptake in the uterine cavity and there were multiple lymph nodes present in the retroperitoneal and bilateral iliac vascular areas (Fig. 2). After consultation, radiologists and gynecological oncologists all suspected the enlargement of lymph nodes were due to tumor cell infiltration based on the diagnosis of endometrial carcinoma.

**Table 1:** Lists laboratory tests and their results

Tests	Results	Normal values
Blood White blood cells (WBC)	8.87	(3.5–9.5) × 10^9^/L
Blood Red blood cells (RBC)	3.52	(3.8–5.1) × 10^9^/L
Blood platelet (PLT)	435	(125–350) × 10^9^/L
Hemoglobin (Hb)	101	115–150 g/L
Hematocrit (HCT)	0.32	0.35–0.45
Urinary protein	Nagative	Negative
Erythrocyte sedimentation rate (ESR)	47	0–20 mm/hr
C-reactive protein (CRP)	3.51	≤ 0.8 mg/dL
Alanine aminotransferase (ALT)	11	7–40 U/L
aspartate aminotransferase (AST)	16	13–35 U/L
Serum creatinine (SCr)	62	53–97 umol/L
Urea	5.1	2.6–7.5 mmol/L
Carbohydrate antigen 125 (CA125)	16.50	0–35 U/ml
Carbohydrate antigen 199 (CA199)	15.00	0–39 U/ml
Neuron-specific enolase (NSE)	9.36	0–17 ng/ml
Carcinoembryonic antigen (CEA)	1.61	0–5 ng/ml
antinuclear antibody (ANA)	spotted type 1:80	< 1:40
Rheumatoid factor (RHF)	< 20	≤ 20 IU/ml
Total complement activity (CH50)	62	23–62 U/ml
Complement (C3)	1.3	0.85–2 g/L
Complement (C4)	0.286	0.12–0.4 g/L
Immunoglobin G (IgG)	27.4	6.94–16.18 g/L
Immunoglobin G1 (IgG1)	14.6	4.05–10.11 g/L
Immunoglobin G2 (IgG2)	9.95	1.69–7.86 g/L
Immunoglobin G3 (IgG3)	0.532	0.11–0.85 g/L
Immunoglobin G4 (IgG4)	0.649	0.03–2.01 g/L
Immunoglobin A (IgA)	5.16	0.7–3.8 g/L
Immunoglobin M (IgM)	0.867	0.6–2.63 g/L
Immunoglobin E (IgE)	25.8	≤ 100 IU/ml

**Fig. 3:**
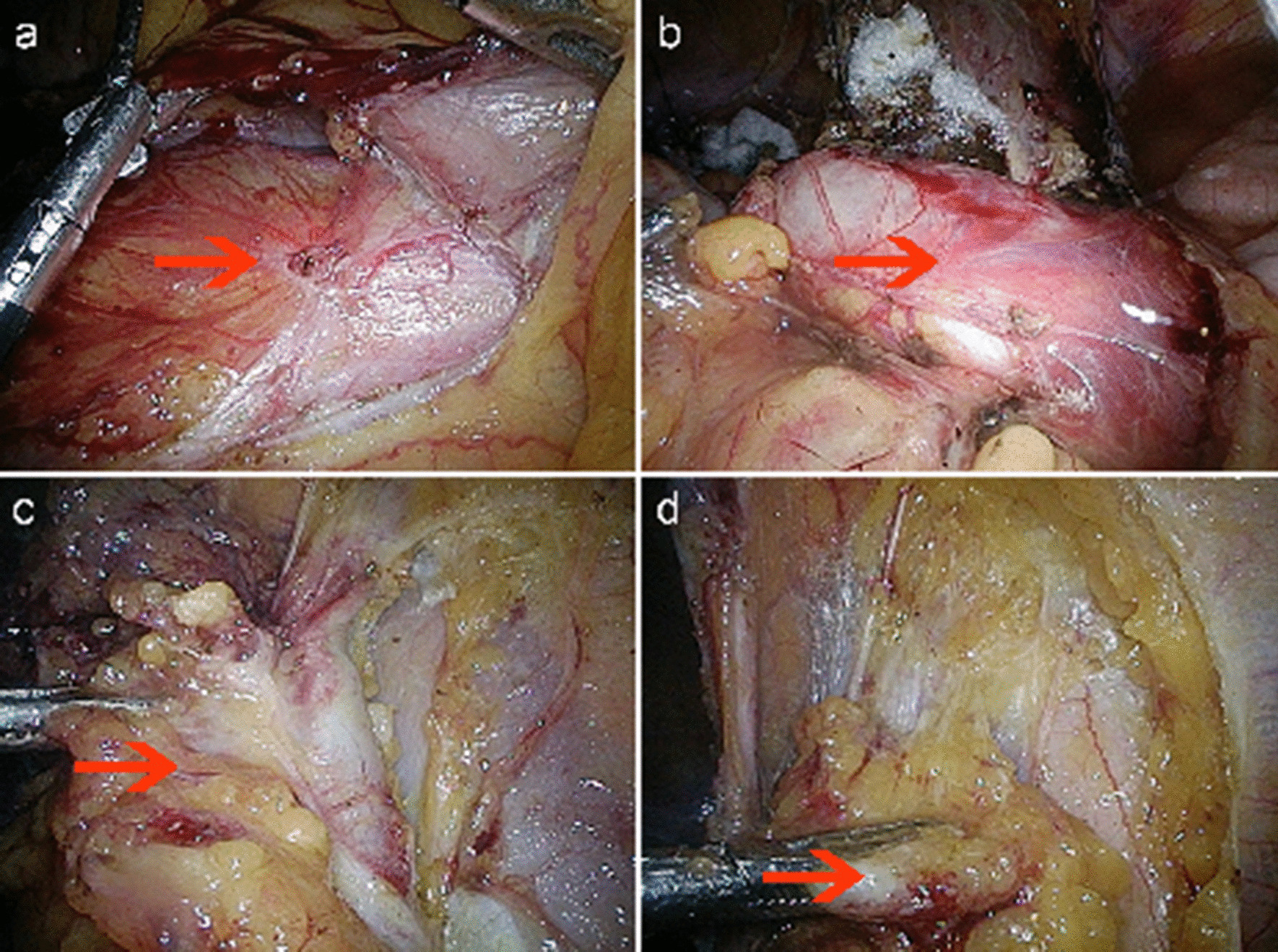
Laparoscopic view during the surgery (the left is the foot side). **a** showed thickened fibrotic peritoneum; **b** showed dilated right ureter; **c** and **d** showed enlarged lymph nodes in right iliac vascular area

The patient was recommended for laparoscopic surgical staging including total hysterectomy/ bilateral salpingo-oophorectomy with lymph node dissection. During the operation, as shown in Fig. 3, thickened fibrotic peritoneum, dilated right ureter, and enlarged lymph nodes could be observed clearly. Notably, because of severe peritoneal fibrosis, the surgery became very difficult, and the risks of bleeding and damage to the surrounding organs were increased, especially when removing the lymph nodes. As a result, we had to perform excision of suspicious and enlarged lymph nodes in the pelvic and aortic regions to verity nodal metastasis. Biopsy samples were taken from the thickened fibrotic retroperitoneum during surgery.

**Fig. 4:**
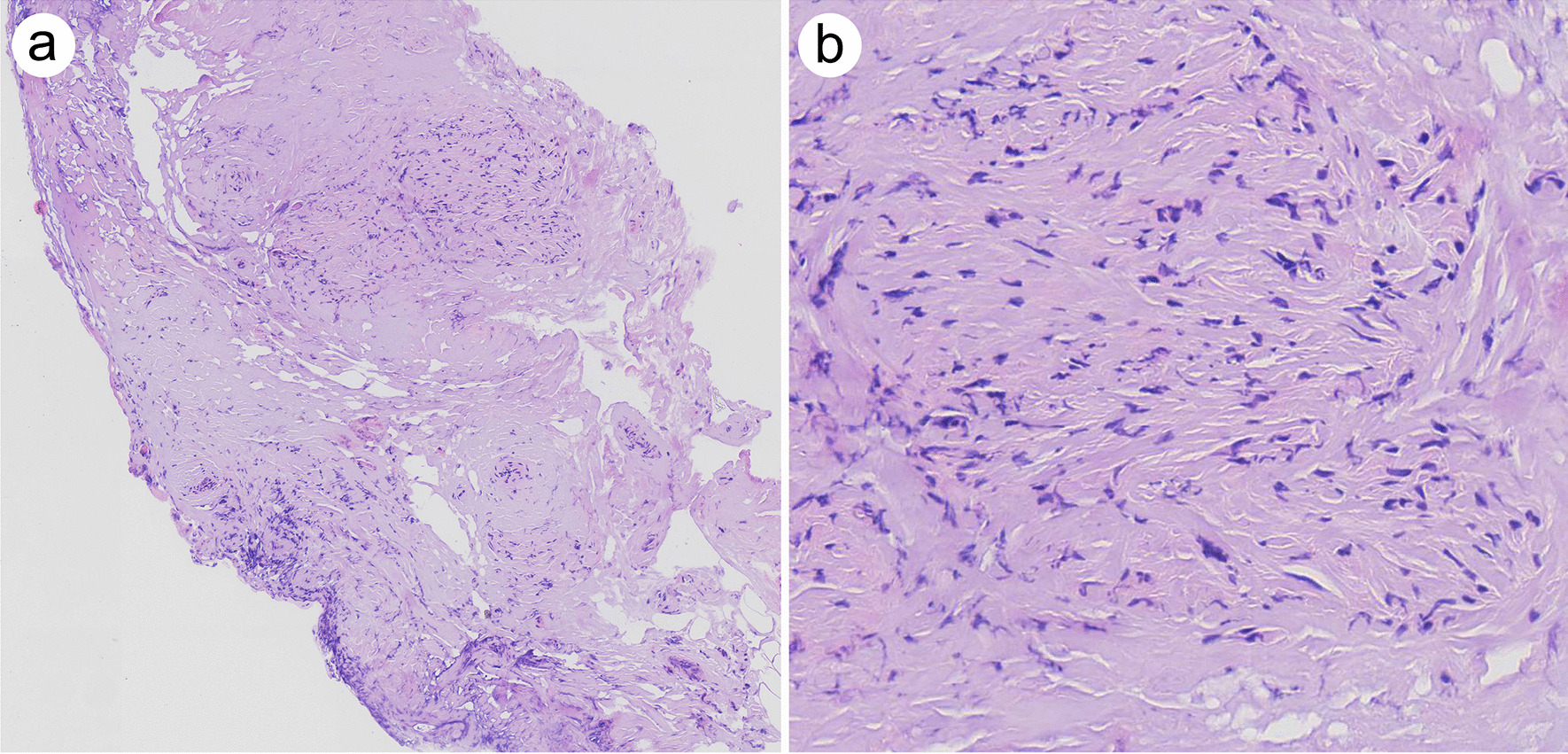
Histopathologic appearance of the retroperitoneum biopsy in this iRPF patient. **a** Low-power magnification view of the retroperitoneum showed fibrosis (**a**, 4 ×); **b** High-power magnification view of the retroperitoneum showed scattered and focal lymphocytes, monocytes and plasma cells infiltration (**b**, 40 ×)

**Fig. 5:**
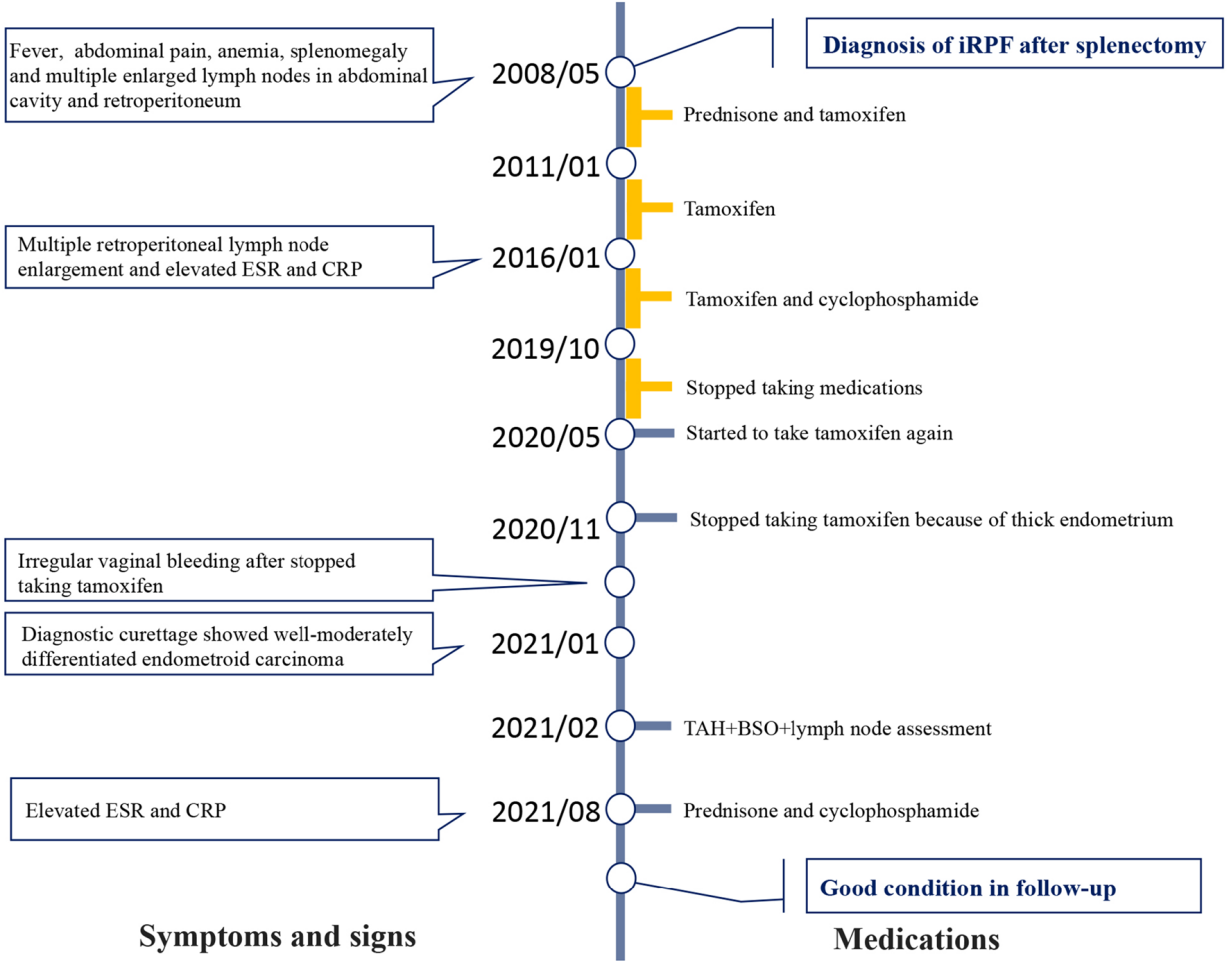
Timeline of historical clinical events and treatment in this patient

Postoperative pathology revealed high-moderately differentiated endometrioid adenocarcinoma, with a size of 1.7 × 1.5 × 1.2 cm, invading the superficial muscle layer (< 1/2 muscle layer) with no vascular invasion. All lymph nodes removed including obviously enlarged lymph nodes showed no evidence of metastasis (left pelvic 0/3, right pelvic 0/4, para-aortic 0/3), but fibrosis was visible. The biopsy of the retroperitoneum showed fibrosis, scattered and focal lymphocytes, monocytes, and plasma cells infiltration (Fig. 4). IHC of IgG4 was negative. According to the International Federation of Gynecology and Obstetrics (FIGO) surgical staging systems for endometrial cancer, this patient was surgically staged as stage IA without adverse risk factors (i.e. age, positive LVSI, tumor size, depth of invasion, and lower uterine [cervical/glandular] segment involvement). After the discussion by multi-disciplinary team, this patient was not recommended to have adjuvant therapy, but close follow-up should be done at regular intervals and simultaneously she was referred to the rheumatology for further maintenance treatment for iRPF. At 3-month and 6-month follow-up in gynecology clinic, no disease recurrence was found. In addition, half a year after the gynecologic surgery, the patient was prescribed prednisone and cyclophosphamide for the treatment of iRPF because of her elevated ESR and CRP. At the time of drafting this paper, the patient was in good condition (Fig. 5).

## Discussion and conclusions

This case report describes a female iRPF patient that was later diagnosed with endometrial cancer. At the time of admission, the patient has a 13-year history of iRPF. RPF is clinically rare with an age-standardized incidence of 0.1 cases/100,000 persons per year [6]. Around 70% of RPF cases are idiopathic with no clear etiology. While the remainder are secondary to certain medications, such as inflammatory disorders, malignant diseases, radiotherapy and abdominal surgery etc. [7]. RPF commonly occurs in adults between the ages of 40 and 60 years old, especially in men (incidents occurring nearly two to three-fold more than female) [2, 8]. It is characterized by a fibroinflammatory tissue surrounding the abdominal aorta and compressing the retroperitoneal organs especially the ureters. Urologic manifestations included obstruction of the ureter, hypertension secondary to renal artery stenosis, renal insufficiency or failure, and non-functioning kidneys are common in cases of ureteral involvement [1, 2]. Laboratory findings of elevated inflammatory markers of ESR and CRP, and reduced Hb often herald the active state of RPF. Studies reported that 10–20% of RPF cases were ANA-positive, and a higher frequency of ANA positivity (30–40%) was observed in iRPF [9, 10]. Recent studies have proposed that iRPF belonged to IgG4-related diseases (IgG4-RD) which manifests as significantly elevated serum IgG4 level and mass-like lesions which could easily be misdiagnosed as tumors [11]. However, the specificity of serum IgG4 level is limited. Liao and colleagues compared the differences between IgG4-RPF and iRPF in a Chinese population which found elevated serum IgE concentration and tissue eosinophilia in the IgG4-RPF subgroup [12]. For this patient, her total IgG was 27.4 g/L, with elevations in IgG1 and IgG2, while IgG3 and IgG4 were normal (Table 1). Her serum IgE was also in normal level of 25.8 IU/ml. Additionally, IHC of her biopsied fibrotic retroperitoneum showed no IgG4-related pathologic features. Therefore, this case was most likely not IgG4 related-RPF.

Imaging examinations played vital roles in the diagnosis and follow-up of RPF. This mainly included ultrasound, CT, MRI, and PET, and in which this disease often manifested as a homogeneous, well-defined plaque wrapping retroperitoneal organs. Differential diagnosis should be made between iRPF and other disease like malignant tumors, infectious diseases, systemic issues and etc. CT is typically the modality of choice to visualize the location and the extent of fibrosis and the possible etiology. Bakir et al. evaluated the role of MRI in RPF which found apparent diffusion coefficient of inactive RPF was higher than that of active RPF or malignant RPF, and diffusion-weighted imaging could contribute to the differentiation of inactive RPF from malignant neoplasms [13]. Moroni et al. reported that 18F-FDG PET/CT could accurately discriminate active from inactive disease (93.9%) [14]. Notably, aseptic inflammatory processes, infections and malignancies could also increase 18F-FDG uptake. Fernando et al.’s study showed that 18F-FDG PET seemed to be able to distinguish cancer from noncancerous RPF [15]. However, in the present case, multiple enlarged lymph nodes, which were considered as lymph node metastasis through preoperative imaging methods including CT, MRI and PET/CT, were pathologically confirmed as fibrosis. Therefore, imaging methods to distinguish RPF and malignancies still needs further assessment and investigation. The most-definitive diagnostic test for RPF is retroperitoneal biopsy which can be performed under imaging guidance in cases with clinical symptoms and the presentation of retroperitoneal mass. The pathological features of iRPF are fibrosis and chronic inflammatory infiltration which consist of a large number of lymphocytes, plasma cells and the formation of lymphoid follicles.

The treatments of RPF include surgery to relieve compression symptoms and medication to prevent further progression of the inflammation. Drug therapy is suitable for RPF patients with early mild symptoms or in late stage of inoperability, or as prevention of postoperative recurrence. Glucocorticoid is currently considered to be one of the most effective drugs for the treatment of RPF. It is rapidly effective and can fully inhibit the early inflammatory response of RPF. However, long-term use of glucocorticoid has many adverse systemic effects and increases the risk of cardiovascular events. Tamoxifen (TMX) was reported to be a suitable alternative to glucocorticoid with no or mild side effects for RPF, especially to those patients that may not be able to tolerate steroids-related toxicity or have contraindications to glucocorticoid [4, 5, 16]. TMX is a non-steroidal anti-estrogen drug which has potential anti-angiogenesis and anti-fibrotic properties [16]. Vaglio and fellow researchers found that receiving TMX for 8 months was significantly less effective in prevention of relapse than 8-month treatment with prednisone in iRPF patients through performing a randomized controlled trial [3]. In addition, TMX has weak estrogen-like effect, long-term use (especially for more than 5 years) may increase the risk of endometrial hyperplasia or even endometrial cancer. Studies had demonstrated that the risk of endometrial cancer was increased following TMX therapy for women with breast cancer (36/6101 vs. 15/6131), predominantly in women over the age of 50 years. However, existing research have not reported any RPF patients that suffered from an increased risk of endometrial cancer when taking TMX [4, 5, 16]. It may in part be explained by the small number of female RPF patients included and limited follow-up period recorded in these studies. Interestingly, the patient mentioned in this study had endometrial cancer after her TMX treatment for iRPF, but the causal link was unknown. Therefore, the risk–benefit ratio and long-term safety of TMX treatment in female RPF patients should be carefully assessed in future studies. A close follow-up including inflammatory markers (ESR and CRP), creatinine, and imaging (ultrasound, CT, MRI or PET) should be advised to monitor the therapeutic response and allow early detection of relapse in RPF patients. Researchers had reported that the relapse rates of RPF varied from 22 to 69% [3, 8, 17]. Therefore, long-term monitoring and remission maintenance strategies must be considered.

For this case, there are some key points that could be taken away. The diagnosis of RPF remains challenging due to the nonspecific clinical symptoms and the lack of standardized diagnostic criteria. Imaging helped to make diagnosis but there were many limitations when it came to differentiating between iRPF and other malignancies. Considering this, biopsy may be the best way to obtain a reliable diagnosis. This patient was diagnosed with iRPF after a splenectomy. Long-term use of tamoxifen might have been a high-risk factor of endometrial cancer. Accurate diagnosis benefited from the careful history taking and thorough physical examination. Organ involvements were evaluated before surgery. Her right kidney almost lost all function from long-lasting obstructive uropathy, but the left kidney compensated so she had almost normal renal function, and this was enough so she could tolerate surgery. Imaging examinations including CT, MRI and PET/CT all showed enlarged lymph nodes and increased FDG uptake in PET/CT. Clinicians believed that it was lymph node metastasis based on patients’ history and imaging findings. However, these enlarged lymph nodes were surgically proved to have no cancer involvement. This case report stressed the difficulty to distinguish between lymph node metastasis and inflammatory hyperplasia by imaging methods. Here, lymph node assessment with biopsy seemed to be particular important. For early-stage endometrial cancer, studies had shown that systematic lymphadenectomy did not benefit in overall survival but improved surgical staging [18, 19]. Thus, the clinical benefit of routine lymphadenectomy should be further investigated to avoid additional surgical complications and over-treatment. Criteria of < 50% myometrial invasion, tumor size < 2 cm, and G1-2 differentiated endometrioid histology were suggested as indication of low-risk for nodal metastases with endometrioid uterine cancer [20]. Further large data studies aiming to see if multiple biopsies or molecular markers may be useful to differentiate lymph node inflammatory hyperplasia from tumor metastasis in RPF patients. However, for those patients with clinically enlarged or suspicious lymph nodes in cancer patients, lymph nodes still should be resected to rule out metastatic disease. Additionally, long-term use of TMX may increase the risk of endometrial cancer. Therefore, TMX should be carefully considered especially in postmenopausal females, and close monitoring should be performed regularly to aid early detection of gynecological diseases.

## Data Availability

All data generated or analyzed during this study are included in this published article.

## Abbreviations

RPF: Retroperitoneal fibrosis
iRPF: Idiopathic retroperitoneal fibrosis
Hb: Hemoglobin
ESR: Erythrocyte sedimentation rate
ANA: Antinuclear antibody
CRP: C-reactive protein
CTU: CT urography
FDG: Fluorodeoxyglucose
IHC: Immunohistochemistry
FIGO: International Federation of Gynecology and Obstetrics
IgG4-RD: IgG4-related diseases
TMX: Tamoxifen
